# Whole-Genome Analysis of Human Influenza A Virus Reveals Multiple Persistent Lineages and Reassortment among Recent H3N2 Viruses

**DOI:** 10.1371/journal.pbio.0030300

**Published:** 2005-07-26

**Authors:** Edward C Holmes, Elodie Ghedin, Naomi Miller, Jill Taylor, Yiming Bao, Kirsten St George, Bryan T Grenfell, Steven L Salzberg, Claire M Fraser, David J Lipman, Jeffery K Taubenberger

**Affiliations:** **1**Center for Infectious Disease Dynamics, Department of Biology, Pennsylvania State University, University Park, Pennsylvania, United States of America,; **2**Institute for Genomic Research, Rockville, Maryland, United States of America,; **3**Wadsworth Center, New York State Department of Health, Albany, New York, United States of America,; **4**National Center for Biotechnology Information, National Library of Medicine, National Institutes of Health, Department of Health and Human Services, Bethesda, Maryland, United States of America,; **5**Department of Molecular Pathology, Armed Forces Institute of Pathology, Rockville, Maryland, United States of America; Princeton UniversityUnited States of America

## Abstract

Understanding the evolution of influenza A viruses in humans is important for surveillance and vaccine strain selection. We performed a phylogenetic analysis of 156 complete genomes of human H3N2 influenza A viruses collected between 1999 and 2004 from New York State, United States, and observed multiple co-circulating clades with different population frequencies. Strikingly, phylogenies inferred for individual gene segments revealed that multiple reassortment events had occurred among these clades, such that one clade of H3N2 viruses present at least since 2000 had provided the hemagglutinin gene for all those H3N2 viruses sampled after the 2002–2003 influenza season. This reassortment event was the likely progenitor of the antigenically variant influenza strains that caused the A/Fujian/411/2002-like epidemic of the 2003–2004 influenza season. However, despite sharing the same hemagglutinin, these phylogenetically distinct lineages of viruses continue to co-circulate in the same population. These data, derived from the first large-scale analysis of H3N2 viruses, convincingly demonstrate that multiple lineages can co-circulate, persist, and reassort in epidemiologically significant ways, and underscore the importance of genomic analyses for future influenza surveillance.

## Abstract 2

Evolution of the flu virus is analyzed via genomic phylogeny; humans are found to provide a reservoir of antigenic variability implicit in flu adaptation and virulence.

## Introduction

Influenza A viruses are negative-strand RNA viruses of the Family Orthomyxoviridae that infect a wide variety of warm-blooded animals, including domestic and wild birds and mammals (e.g., humans, pigs, and horses). The natural reservoir for influenza virus is thought to be wild waterfowl, and genetic material from avian strains episodically emerges in strains infectious to humans. These human viruses continually circulate in yearly epidemics (mainly during the winter months in temperate climates), and antigenically novel strains emerge sporadically as pandemic viruses [1,2]. In the United States, influenza is estimated to kill 30,000 people in an average year [3,4]. Every few years, influenza epidemics boost the annual mortality level above this average, causing 10,000–15,000 additional deaths. Occasionally, and unpredictably, global pandemics of influenza occur, infecting 20% to 40% of the population in a single year and raising death rates dramatically above normal levels. Pandemic influenza A viruses emerged three times during the last century: in 1918 (H1N1 subtype), in 1957 (H2N2), and in 1968 (H3N2) [2,5]. The recent circulation of highly pathogenic avian H5N1 viruses in Asia from 2003–2005 has caused at least 52 human deaths [6,7] and has raised concern about the development of a new pandemic [5]. How and when novel influenza viruses emerge as pandemic strains and their precise mechanisms of pathogenesis are still not understood.

While the risk of pandemic influenza poses a significant public health concern, inter-pandemic or epidemic influenza remains a major cause of morbidity and mortality. The influenza A surface glycoprotein hemagglutinin (HA) protein is under selective pressure for change in order to evade the host's immune system [8]. Antibodies against the HA protein inhibit receptor binding and are very effective at preventing reinfection with the same strain. However, HA can change to evade previously acquired immunity either by antigenic drift, whereby mutations of the currently circulating HA gene disrupt antibody binding, or by antigenic shift, in which the virus acquires an HA of a new subtype by reassortment of one or more gene segments. While it is generally accepted that drift is responsible for inter-pandemic influenza outbreaks and shift for pandemics, there are exceptions to this rule. For example, in 1977, an H1N1 virus re-emerged but failed either to cause a pandemic or to replace the prevailing H3N2 subtype [9].

The importance of predicting the emergence of new circulating influenza strains for subsequent annual vaccine development cannot be underestimated [10]. To this end, the global influenza surveillance network coordinated by the World Health Organization was established to select the candidate strains of influenza A and B for the yearly production of influenza vaccine in both the northern and southern hemispheres. The network characterizes the antigenic properties of influenza viruses using HA inhibition assays and sequencing of the HA1 domain (globular head) of HA of a select number of strains [11,12]. Antigenic, genetic, and epidemiologic data are then examined to make recommendations of candidate vaccine strains.

A number of retrospective studies have been performed using partial HA gene sequences to understand, and sometimes predict, the evolution of human H3N2 strains [13–17]. Accumulation of amino acid replacements in HA is clustered in five variable antigenic sites [18] around the receptor binding site. Phylogenetic analysis has revealed that 18 codons in the HA1 domain exhibit significantly more nonsynonymous nucleotide substitutions than synonymous ones, constituting a signature of strong, selectively driven, antigenic drift [15]. More recently the antigenic and genetic evolution of HA was compared through the construction of an antigenic map of H3N2 viruses [17]. Although antigenic evolution was found to be more punctuated than genetic evolution over the same time period, the two measures of HA drift were generally correlated.

Despite the wealth of data on the molecular evolution of influenza viruses, how the entire genome of influenza A virus evolves during epidemic years is unclear, particularly as past sample sizes have been inadequate. While antigenic drift of HA is clearly of vital importance in the survival of an influenza strain, other factors, including HA receptor binding specificity [19], antigenic drift of neuraminidase (NA) [20], matched activity between HA and NA [21–23], and the interaction of the other influenza proteins with each other and their host cells, are all likely to affect viral fitness in a polygenic manner. Similarly, it is unclear how many lineages of influenza A viruses persist between seasonal epidemics, particularly in genes other than that encoding HA.

To this end the National Institute of Allergy and Infectious Diseases of the National Institutes of Health has funded the Influenza Genome Sequencing Project with several partners [24,25] including the Institute for Genomic Research. To date, 156 genomes of human H3N2 viruses collected between 1999 and 2004 from New York State have been completely sequenced and deposited in GenBank. We have performed an initial analysis of these viruses and have found evidence for both the existence of multiple clades of viruses co-circulating at the same time point and for multiple reassortment events among these clades. One of these reassortment events was the likely progenitor of the A/Fujian/411/2002-like drift epidemic of the 2003–2004 influenza season, in which there was a poor match between the vaccine strain and the predominant circulating viruses of that year [26,27]. This report extends recent observations of reassortant H3N2 influenza A viruses from the southern hemisphere during this same time period [28].

## Results

### Analysis of the Concatenated Complete Genome of the New York State Influenza Isolates

**Figure 1:**
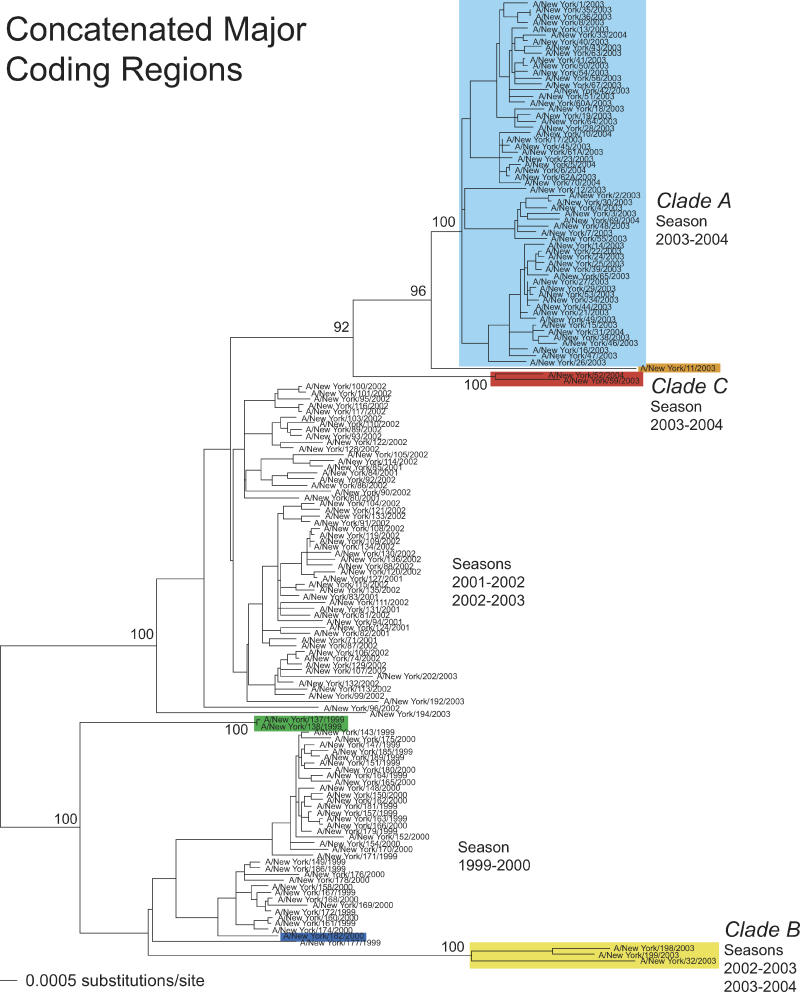
Evolutionary Relationships of Concatenated Major Coding Regions of Influenza A Viruses Sampled in New York State during 1999–2004 The maximum likelihood phylogenetic tree is mid-point rooted for purposes of clarity, and all horizontal branch lengths are drawn to scale. Bootstrap values are shown for key nodes. Isolates assigned to clade A (light blue), clade B (yellow), and clade C (red) are indicated, as are those isolates involved in other reassortment events: A/New York/11/2003 (orange), A/New York/182/2000 (dark blue), and A/New York/137/1999 and A/New York/138/1999 (green).

Three major clusters of sequences were apparent in phylogenetic trees of the 156 complete genomes of H3N2 influenza A viruses sampled from New York State. These corresponded to particular influenza seasons (winter months): (a) 1999–2000, (b) 2001–2002 and 2002–2003 together (although only five members of latter season are present in these data), and (c) 2003–2004 (Figure 1). Such temporal structure is commonly observed in trees of influenza A virus, and this is thought to be largely driven by positive selection acting on the HA gene [17]. However, a number of isolates did not fall into these three groups. First, three isolates from the 2002–2003 and 2003–2004 seasons—A/New York/32/2003, A/New York/198/2003, and A/New York/199/2003—formed a distinct phylogenetic group whose closest relationship was with those viruses sampled in the 1999–2000 season, rather than with those sampled during later seasons. We have denoted the two groups of viruses circulating after the 2001–2002 season as clade A (the major cluster after the 2001–2002 season) and clade B (the minor cluster comprising isolates 32, 198, and 199). Second, isolates A/New York/52/2004 and A/New York/59/2003 (designated as clade C in Figure 1) occupied a position intermediate between clade A and the 2001–2002 group of viruses, as did isolate A/New York/11/2003. Finally, isolates A/New York/137/1999 and A/New York/138/1999 formed a group that was divergent from the main group of viruses sampled in the 1999–2000 season.

### Analysis of Individual Gene Segments

**Figure 2:**
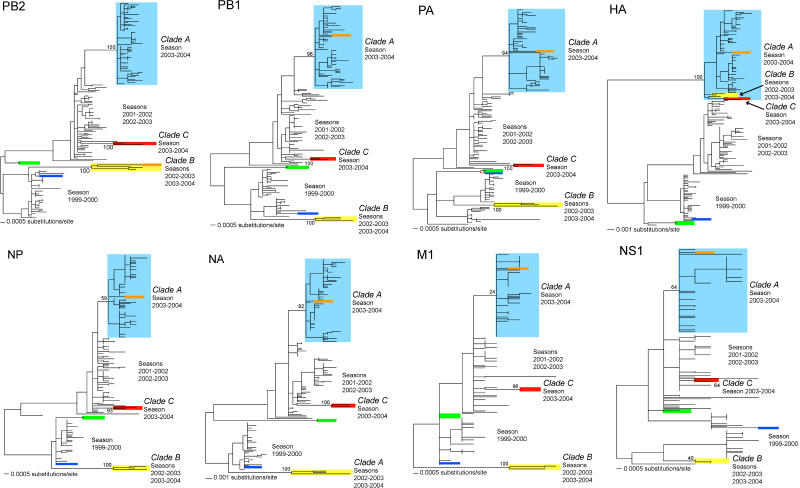
Evolutionary Relationships of Individual Major Coding Regions from Influenza A Viruses Sampled in New York State during 1999–2004 All maximum likelihood phylogenetic trees are mid-point rooted for purposes of clarity only, and all horizontal branch lengths are drawn to scale. Bootstrap values are shown for clades A, B, and C. Colors are as in Figure 1.

To investigate the evolutionary history of the outlier viruses in more detail we inferred phylogenetic trees for each of the eight individual gene segments (Figure 2). Strikingly, although the distinction between clades A, B, and C was apparent in seven of the eight genes, no such separation was seen in the HA phylogeny. In this case, clades B and C clearly clustered within clade A and with strong bootstrap support. The close phylogenetic relationship of these three groups of viruses in HA set against a background of genetic divergence in all other segments strongly suggests that these data contain evidence for at least two independent reassortment events, one involving clades A and B and another involving clade C and either clade A or clade B. In the case of the clade C viruses, the separate gene phylogenies also reveal that these isolates share a common ancestry with viruses first sampled during the 2001–2002 season, while the clade B viruses share a closer relationship with those viruses of the 1999–2000 season.

Two more major phylogenetic displacements suggestive of reassortment involving other segments were similarly identified. First, isolate A/New York/11/2003, which fell within clade A in seven of the gene trees (including HA), clustered with clade B viruses in PB2. Consequently, isolate A/New York/11/2003 represents a reassortment of two segments between clades A and B. Second, isolate A/New York/182/2000, which clustered with the main set of viruses sampled during the 1999–2000 season in most of the gene trees, was very closely related to the divergent A/New York/137/1999 and A/New York/138/1999 isolates in PA and M1, although the high degree of genetic similarity among all viruses in M1 precludes a further analysis of reassortment in this case.

**Figure 3:**
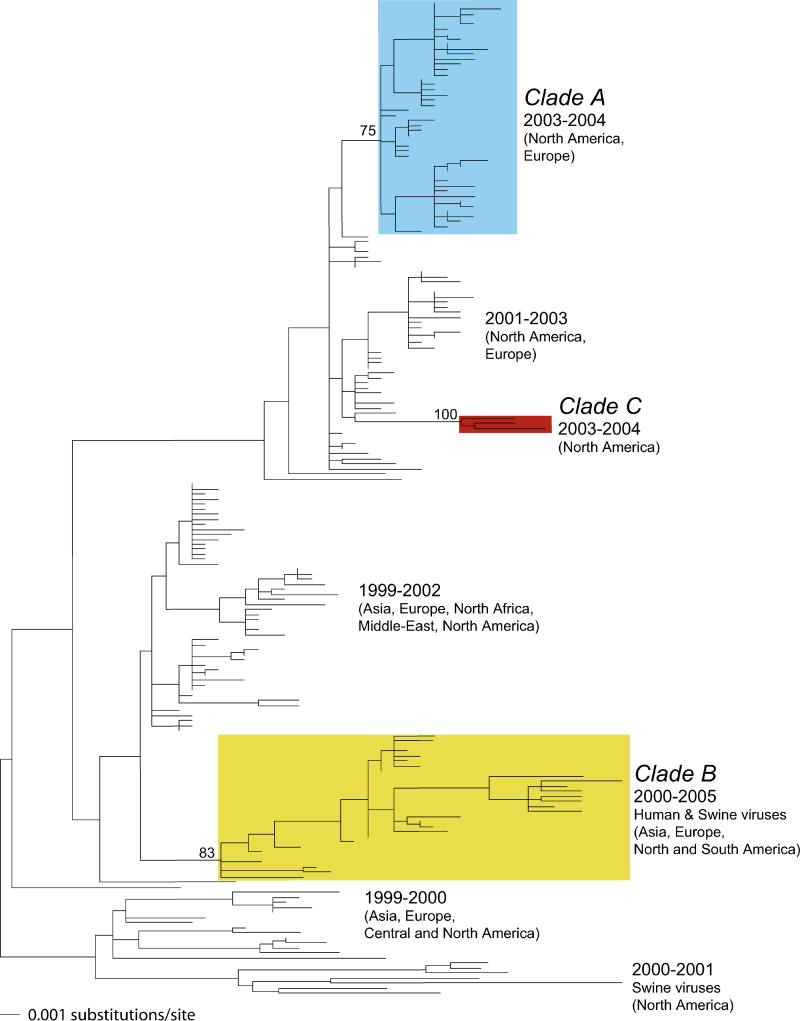
Evolutionary Relationships of 197 NA Sequences Sampled from Mammalian Hosts during 1999–2005 The maximum likelihood phylogenetic tree is mid-point rooted for purposes of clarity only, and all horizontal branch lengths are drawn to scale. Bootstrap values are shown for clades A, B, and C. Colors are as in Figure 1.

To determine the direction of the reassortment events in HA, we inferred phylogenetic trees of larger datasets comprising the New York State isolates and representatives of the other human and swine H3N2 viruses sampled during the same time period. Because sequences from the core genes have only been sporadically collected, this analysis necessarily focused on HA and NA. As expected from the phylogenetic analysis of the New York State viruses, the distinction between clades A, B, and C was apparent in the NA gene tree (Figure 3) as well as the core genes (trees shown in Figure S1). Moreover, in the NA gene tree, viruses sampled from a variety of locations during 2000–2005 fell into clade B, including Europe (Denmark and Norway), Asia (China and Singapore), and the Americas (Argentina, Brazil, and the United States). Hence, although clade B was at low frequency in the New York State dataset, it represents a distinct lineage of H3N2 viruses globally circulating from at least 2000 to 2005. Similarly, a third North American virus, A/Charlottesville/03/2004, was designated as clade C.

**Figure 4:**
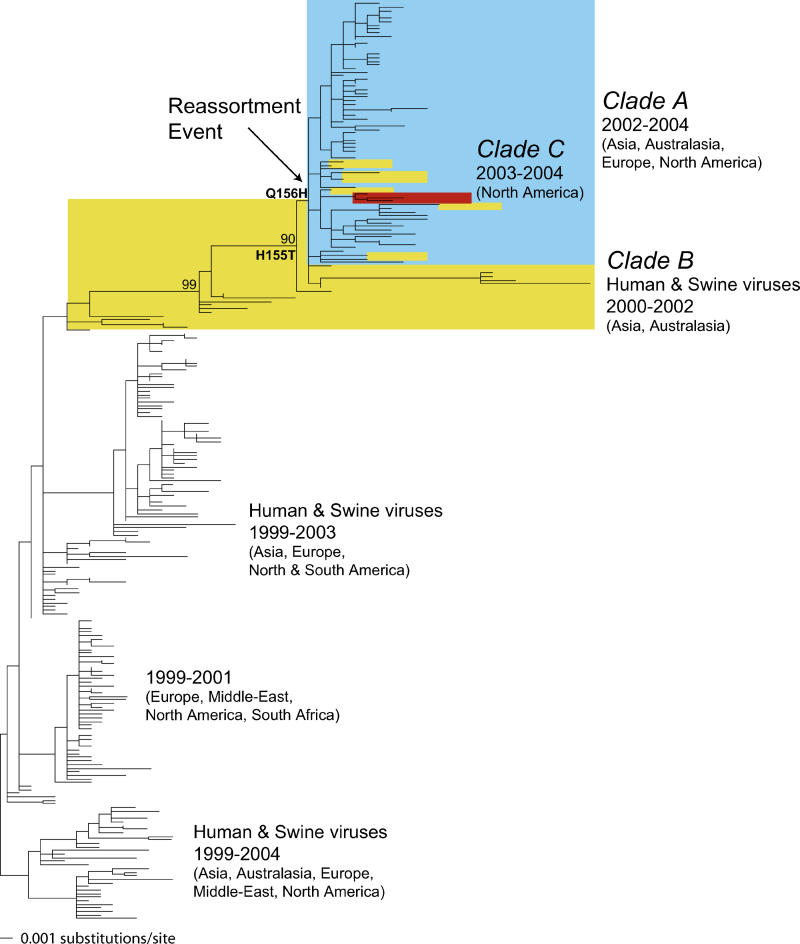
Evolutionary Relationships of 256 Sequences of the HA1 Sequences Sampled from Mammalian Hosts during 1999–2004 The maximum likelihood phylogenetic tree is rooted on a divergent set of human and swine viruses for purposes of clarity only, and all horizontal branch lengths are drawn to scale. Bootstrap values are shown for key nodes. Colors are as in Figure 1, with yellow indicating all those viruses identified as clade B viruses from the analysis of the expanded NA dataset. The phylogenetic location of the reassortment event between clades A and B was set at the position of the most basal clade A virus, defined on the basis of the NA analysis, within the older clade B. The phylogenetic locations of the two critical antigenic mutations at sites 155 and 156 are also shown.

A very different evolutionary history was revealed in HA. In this case, clade A of the New York State viruses expanded to contain the majority of viruses sampled after 2002 and from a variety of locations (Asia, Australasia, Europe, and North America), as well as a number of Asian viruses from 2002 (Figure 4). This large group of viruses then clustered within clade B, such that most clade B viruses sampled from 2000 to 2002 formed a clear, but closely related, outgroup to the later clade A viruses, with the remaining clade B viruses falling within clade A (Figure 4). The three clade C viruses also fell within this expanded clade A. Such a phylogenetic pattern strongly suggests that the HA from both the clade A and clade C viruses was acquired from that present in clade B through reassortment. In this context it is of interest to note that the HA of the A/Fujian/411/2002 virus and related isolates are present in this reassorted clade. Further, the fact that the clade A and B isolates closest to the phylogenetic location of the reassortment event were both sampled in 2002 suggests that the reassortment occurred in this year, although pinpointing the exact phylogenetic location of the recombinant event is difficult given the relatively small number of samples available from this critical time period. Similarly, the fact that these viruses had Asian origins is also compatible with the reassortment event occurring in this region, a hypothesis also supported by a recent analysis of comparable partial genomic analysis of H3N2 isolates from the southern hemisphere [28].

### Analysis of the Coding Differences between Clades A, B, and C

Because both clade A and clade B contain viruses sampled on a near global basis, it is important to determine possible phenotypic differences between them. Table 1 shows the amino acid replacements that consistently distinguish the clade A and B viruses. These changes are not uniformly distributed among the seven segments (not including HA). Of the 48 amino acid differences between clades A and B, 14 fall in NA and nine in NP. PB1, PB2, and PA have five differences each, while M2 and NS1 have three differences, and M1 and NS2 have two. In contrast, there are only nine amino acid differences between the clades A and C: PB2—T9N; PA—A20T, L226I, and N272D; NP—G450S; NA—H40Y and V263I; and NS1—A56T and N143T.

**Table 1:**
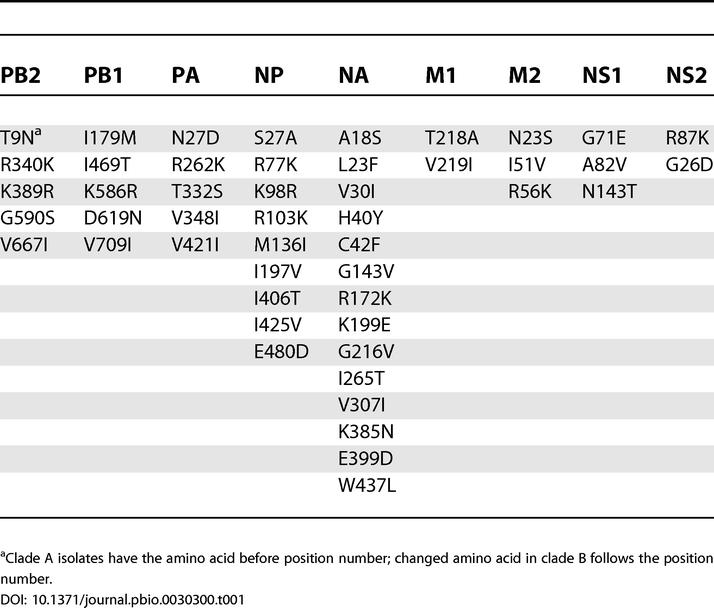
Amino Acid Changes Between the Proteins Encoded by the Clade A and Clade B Viruses

## Discussion

Our analysis of whole genomes of H3N2 influenza A viruses sampled during 1999–2004 has identified two key evolutionary patterns. First, although the majority of viruses isolated after 2002 fall into a single phylogenetic group (clade A), multiple, co-circulating viral lineages are present at particular time points. The genetic diversity of influenza A virus is therefore not as restricted as previously suggested, particularly when genes other than that encoding HA are analyzed. This co-circulation of lineages is most apparent with the identification of three clades of H3N2 viruses that appear to infect the same populations until 2002, after which they acquired a common HA gene through reassortment. Second, and more dramatically, these multiple, co-circulating lineages may have complex genealogical histories and interact through reassortment. Indeed, we have documented two reassortment events involving the HA gene of clade B: one in which it was acquired by the clade A viruses and another in which it was independently acquired by those isolates assigned to clade C. Two further reassortment events involving the PB2 and PA genes were also evident from our phylogenetic analysis. Given that we are only able to reliably detect reassortment when it is associated with major changes in tree topology, it is likely that reassortment among closely related lineages is also commonplace in influenza A viruses.

Reassortment between influenza A viruses has been described in both human and animal viruses [1,29]. Notably, antigenic shift by reassortment between human and avian influenza A viruses has been documented in the formation of the 1957 H2N2 and 1968 H3N2 pandemics [30–32]. Other recent examples of reassortment between human and animal influenza A viruses have resulted in the emergence of novel H3N2 and H1N2 swine viruses in North America and Europe [33,34] and the evolution of H5N1 viruses in Asia from 1997 to the present [35]. Reassortment between co-circulating lineages of human influenza A and more recently influenza B viruses following mixed infection has also been described [36–41]. For example, human H2N2 viruses formed two distinct clades in the 1960s prior to the emergence of the 1968 H3N2 pandemic virus, with one virus a reassortant containing genes of both clades [42]. Similarly, the early H3N2 viruses (1968–1972) acquired the H3 HA and the PB1 gene via reassortment with an avian virus [30,31]. Reassortment between H3N2 and H2N2 viruses may therefore have assisted successful cross-species transmission [42].

Reassortant viruses were also described following the re-emergence of the H1N1 subtype in 1977 that did not replace the previously circulating H3N2 viruses. In this case, co-circulation of influenza viruses of both subtypes continued, and co-infection with both subtypes was reported [43]. While reassortant H3N1 strains were not isolated, H1N1 strains containing reassorted internal protein-encoding gene segments from H3N2 viruses were observed [44,45]. Occasional isolates of H1N2 viruses were also detected after the re-emergence of H1N1 [46,47]. More recently, the widespread circulation of viruses with the H1N2 subtype has been documented [41]. These viruses contained the HA segment of contemporary H1N1 viruses reassorted onto an H3N2 background, a 7:1 reassortment pattern similar to that observed with the sporadically circulating H1N2 viruses of the 1980s and early 1990s [47] and to the dominant reassortments described in this analysis. Since the H1 and N2 subtype proteins were antigenically and genetically similar to co-circulating H1N1 and H3N2 subtype viruses, the emergence of this new subtype did not result in an epidemiologically significant event [41]. Reassortment among co-circulating clades of H3N2 viruses like that observed in the current study has also been previously described, including reassortment of the NA gene segment [48] and the core protein-encoding segments [49].

Most prior phylogenetic studies of human influenza A have suggested that inter-pandemic evolution may be essentially described as a series of successions by variants of the previous season's dominant strain. These successions are largely determined by strong positive selection acting on the abundance of mutational diversity in the HA of the dominant strain. However, we found that at least four reassortment events occurred among human viruses during the period 1999–2004 and that two of these involved a major change in HA. Recently, Barr et al. independently provided phylogenetic evidence of the clade A–clade B reassortment described here in an analysis of predominantly southern hemispheric influenza A H3N2 isolates collected during the same period [28]. To our knowledge, these analyses are the first demonstrations of the emergence of a major antigenically variant virus derived by reassortment between two distinct clades of co-circulating H3N2 viruses rather than by antigenic drift. These findings suggest that the ongoing evolution of human influenza A virus is likely to be more complex than depicted in standard models of antigenic drift; multiple lineages of antigenically distinct viruses can persist within populations and, through their reassortment, produce major changes in antigen space. Similarly, the persistence of multiple lineages of H3N2 within a single population indicates that human populations represent a larger reservoir of genetically distinct viruses than previously anticipated. Indeed, it is possible that key changes in antigen type, depicted as jumps between cluster types [17], could be strongly influenced by reassortment among co-circulating human strains. Crucially, the real importance of both lineage persistence and reassortment in influenza A virus evolution could not be determined until a representative sample of full-genome sequences was collected from a single population.

In the 2003–2004 influenza season, a major drift variant emerged in both the northern and southern hemispheres [50–52]—the A/Fujian/411/2002-like variant. In the United States, the 2001–2002 influenza was an H3N2-predominant season, and all antigenically characterized isolates matched the A/Moscow/10/1999 vaccine strain [53]. In the 2002–2003 season, which was an H1- and influenza B–dominant season in the United States, a minority of antigenically characterized H3N2 isolates were different from the Moscow/10/1999-like vaccine strain [54], probably coinciding with the emergence of the Fujian strain. Indeed, in our phylogenetic analysis two of five H3N2 viruses from the 2002–2003 season fall into clade B, along with two newly sequenced isolates that were not included in our analysis (A/New York/201/2003 and A/New York/203/2003). Consequently, clade B viruses made up a significant fraction of the few H3N2 isolates in that season. In contrast, only a small minority of influenza H3N2 viruses sampled in Europe at this time were antigenically characterized as Fujian-like [50], while in the southern hemisphere's 2003 influenza season the Fujian strain was predominant [28,51]. The 2003–2004 influenza season in the northern hemisphere was also dominated by the Fujian strain [55], although the vaccine contained the H3N2 (A/Panama/2007/1999) from the previous year. This strain was a poor antigenic match to the Fujian strain [26,27], which in turn led to reduced vaccine effectiveness. Thirteen amino acid changes distributed across the five antigenic sites of the HA1 domain distinguish the A/Panama/2007/1999-like and A/Fujian/411/2002-like strains. While these replacement changes appear to have phenotypic consequences (e.g., replication efficiency in eggs), only two residues, 155 and 156, are responsible for the major antigenic differences between the strains [27].

Although data are insufficient for precise determination of the timing of these two critical mutations, the available data are most consistent with these changes occurring in a relatively short time period before the reassortment event. The histidine to threonine change at site 155 and the glutamine to histidine change at site 156 are present in all the clade A reassortant isolates as well as in clade B isolates from 2003–2004, thus suggesting that they occurred prior to the reassortment event. No available clade B isolates prior to 2002–2003 have either of these mutations, and we were able to identify only three “intermediate” isolates from 2002–2003 (A/Kwangju/219/2002, A/Kwangju/243/2002, and A/Cheonnam/340/2002) with the replacement at site 155 but not at site 156. Overall, the data presented here, coupled with those recently reported [28], reveal that the HA segment of the H3N2 clade B viruses, present in low frequencies at least since 1999, was reassorted into clade A of H3N2, probably in 2002 soon after the appearance of these two critical mutations, and that this reassortment was central to the production of antigenically variant strains that were poorly matched to the vaccine strain in the 2003–2004 season [27]. In addition, presumably because of the high rate of reassortment and the fitness advantage conferred by these two mutations, this clade B HA segment also appeared to replace the HA segment of the clade C strains. Finally, though previously present only in low frequencies, recent sequencing of HA and NA by investigators in Denmark [56] and published phylogenetic trees from Australia [28,51] show the existence of clade B virus, suggesting that it continued to have a global distribution and sometimes at appreciable frequency.

Several questions remain unanswered by our study. Since the HA donated by clade B led to a major expansion of the reassorted clade A, it is uncertain why clade B did not initially out-compete clade A without reassortment. One possibility is that the HA of clade B had an intrinsically higher fitness than other HAs circulating at the same time but was unable to reach a high frequency in the New York population owing to linkage to mutations located in other segments that reduced the overall fitness of this genotype. According to this hypothesis, it was not until it was placed by reassortment into a more favorable genetic background, in this case the clade A viruses, that its fitness advantage was realized. Since clade B itself appeared to proliferate in other regions, it will be useful to analyze whole-genome sequence from these isolates when they are available.

More generally, it is clear that the genotypic basis to viral fitness has not been entirely elucidated. In particular, it is likely that interactions among viral proteins and between viral proteins and host factors play a key role. In this respect it is notable that of the 48 amino acid differences that distinguish the clade B viruses, nine fall in NP and 14 fall in NA (see Table 1). In NP, the replacement at residue 425 falls within an HLA-B35-restricted cytotoxic T lymphocyte (CTL) epitope and is not commonly seen in human populations [57]. Two other changes, at residues 27 and 197, have also been identified as being contained in two HLA-A11-restricted CTL epitopes [58]. Another unusual change in NP is M136I: a change to methionine at this site was proposed as one of six human adaptive changes distinguishing the 1918 NP from avian NPs [59]. Indeed, all 45 available NP sequences from human H2N2 viruses, and all but four of approximately 250 human H3N2 NP sequences, maintain this methionine. Only the three New York clade B viruses, as well as A/Taiwan/1/71, have a change to an isoleucine at this residue. Of the changes in NA, six of them lie in five regions previously identified as being phylogenetically important regions, residues 40, 42, 143, 199, 307, and 385 [60], and therefore play a role in virus–host interaction. Three further changes, at residues 172, 199, and 307, map to antigenic sites [20], while a change at residue 18 has been mapped as an HLA-A2-restricted CTL epitope [58]. Similarly, two changes in M2, at residues 51 and 56, map to an HLA-A11-restricted CTL epitope, while residue 82 of NS1 maps to an HLA-A2-restricted CTL epitope [58]. Another change between clade A and B viruses, at residue 226 of PA, also maps to an HLA-A2-restricted CTL epitope [58]. It is possible that some of the mutations that fall in CTL epitopes assist the persistence of clade B by elongating the viral infectious period [61]. However, any combination of the constellation of amino acid changes may have altered the fitness of the clade B viruses in a way that we do not have the ability to understand from sequence analysis alone. Interestingly, Gulati et al. [62] have recently shown that Fujian-like strains have a mismatch in their HA and NA activities that is probably the result of the reassortment event described in the current study. The significance of this for the pathophysiology of the virus is currently unknown.

In summary, our study clearly demonstrates the utility of whole-genome analyses of influenza A viruses, and further makes clear that additional whole-genome analyses are required to understand fully the evolutionary mechanisms and epidemiological dynamics of this virus. While antigenic variance of HA is still the dominant selective pressure on human influenza A virus evolution, the finding that antigenically novel clades emerge by reassortment among persistent viral lineages rather than via antigenic drift is of major significance for vaccine strain selection.

## Materials and Methods

### Influenza viruses used in this study

The influenza virus isolates were collected as part of the diagnostic service provided by the Virus Reference and Surveillance Laboratory at the Wadsworth Center, New York State Department of Health. Viruses were received as part of outbreak investigations, through the reference function of the laboratory, and, since 2001, as part of a sentinel physician influenza surveillance program. Viruses were passaged minimally in primary rhesus monkey kidney cell culture and the RNA extracted from the clarified supernatant. Whole-genome sequence information was derived at the Institute for Genomic Research using methods described elsewhere (E. Ghedin, N. A. Miller, M. Shumway, J. Zaborsky, T. Feldblyum, et al., unpublished data). Use of the diagnostic samples in this study was approved by the New York State Department of Health Institutional Review Board.

### Sequences used in the analysis

Sequence data for 156 complete genomes of influenza A virus (H3N2) sampled from New York State during the period 1999–2004 were downloaded from GenBank (1,248 separate accessions, representing the eight gene segments of 156 individual influenza isolates; GenBank accession numbers available from http://www.ncbi.nlm.nih.gov/genomes/FLU/FLU.html). Separate sequence alignments were then manually compiled for the major coding regions of each segment: PB2, 2,277 bp; PB1, 2,271 bp (PB1 protein); PA, 2,148 bp; HA, 1,698 bp; NP, 1,494 bp; NA, 1,407 bp; MP, 756 bp (M1 protein); and NS, 690 bp (NS1 protein). An alignment of the concatenated major coding regions was also constructed for all 156 isolates (12,741 bp). To place the New York State viruses in the context of global H3N2 evolution, larger datasets were complied comprising the New York State isolates plus all other mammalian influenza A viruses sampled from 1999–2004 available on GenBank and the Los Alamos Influenza Sequence Database (http://www.flu.lanl.gov/). For the six core genes, few such background sequences were available, particularly from clades B and C (see below). However, far larger numbers of background sequences were available for the HA1 domain portion of HA and for sequence of NA genes. To facilitate the computational analysis of these background datasets, those New York State isolates with identical sequences were removed from the analysis. This resulted in alignments of the following sizes: PB2, 134 sequences, 2,277 bp; PB1, 135 sequences, 2,271 bp; PA, 140 sequences, 2,148 bp; HA, 256 sequences, 987 bp; NP, 117 sequences, 1,494 bp; NA, 197 sequences, 1,407 bp; M1, 73 sequences, 756 bp; and NS1, 75 sequences, 690 bp. A full list of the isolates used in this study is provided in Table S1.

### Phylogenetic analysis

Phylogenetic trees were inferred for all of the datasets described above using the maximum likelihood method available in the PAUP* package [63] (see Table S2). In each case the general time-reversible model of nucleotide substitution was used also incorporating a proportion of invariable sites and a gamma distribution of rate variation among sites with four rate categories. All parameter values were estimated from the empirical data and are given in Table S2. Tree bisection–reconnection branch-swapping was used in all cases apart from the expansive (“background”) HA and NA datasets, which contained so many sequences that the analysis was restricted to subtree pruning–regrafting branch-swapping. To assess the reliability of key nodes on the phylogenetic trees, a bootstrap resampling analysis was also undertaken in each case. This involved the inference of 1,000 replicate neighbor-joining trees using the maximum likelihood substitution model inferred for each dataset.

## Supporting Information

Figure S1 Phylogenetic Trees of New York State and Background H3N2 Strains Inferred from the Core Genes Maximum likelihood phylogenetic trees depicting the evolutionary relationships of the remaining six segments (major coding regions only) from the H3N2 influenza A viruses sampled in New York State during the period 1999–2004 (unique sequences only) and the “background” viruses taken from GenBank or the Los Alamos Influenza Sequence Database. All trees are mid-point rooted for purposes of clarity only, and all horizontal branch lengths are drawn to scale. Bootstrap values are shown for clades A, B, and C. Colors are as in Figure 1.
(715 KB EPS).

Table S1 Isolates of Influenza A Virus H3N2 Used in This Study (164 KB DOC).

Table S2 Parameter Values for Maximum Likelihood Phylogenetic Analysis (50 KB DOC).

## Abbreviations

CTL: cytotoxic T lymphocyte
HA: hemagglutinin
NA: neuraminidase
