# Supplementary material for: Whole-Genome Analysis of Human Influenza A Virus Reveals Multiple Persistent Lineages and Reassortment among Recent H3N2 Viruses
Source: PLoS Biol. 2005 Jul 26;3(9):e300. doi: 10.1371/journal.pbio.0030300 (PMC1180517; doi:10.1371/journal.pbio.0030300)
Supplement: Supplementary file 1 [file pbio.0030300.st001.doc]

Supplementary Information 1. Isolates of influenza A virus H3N2 used in this study. All sequences are available on GenBank and/or the Influenza Sequence Database.

1. New York State Isolates

| A/New York/1/2003 | A/New York/161/1999 | A/New York/4/2003 |
| --- | --- | --- |
| A/New York/10/2004 | A/New York/162/2000 | A/New York/40/2003 |
| A/New York/100/2002 | A/New York/163/1999 | A/New York/41/2003 |
| A/New York/101/2002 | A/New York/164/1999 | A/New York/42/2003 |
| A/New York/103/2002 | A/New York/165/2000 | A/New York/43/2003 |
| A/New York/104/2002 | A/New York/166/2000 | A/New York/44/2003 |
| A/New York/105/2002 | A/New York/167/1999 | A/New York/45/2003 |
| A/New York/106/2002 | A/New York/168/2000 | A/New York/46/2003 |
| A/New York/107/2002 | A/New York/169/2000 | A/New York/47/2003 |
| A/New York/108/2002 | A/New York/17/2003 | A/New York/48/2003 |
| A/New York/109/2002 | A/New York/170/2000 | A/New York/49/2003 |
| A/New York/11/2003 | A/New York/171/1999 | A/New York/5/2004 |
| A/New York/110/2002 | A/New York/172/1999 | A/New York/50/2003 |
| A/New York/111/2002 | A/New York/174/2000 | A/New York/51/2003 |
| A/New York/113/2002 | A/New York/175/2000 | A/New York/52/2004 |
| A/New York/114/2002 | A/New York/176/2000 | A/New York/53/2003 |
| A/New York/115/2002 | A/New York/177/1999 | A/New York/54/2003 |
| A/New York/116/2002 | A/New York/178/2000 | A/New York/55/2003 |
| A/New York/117/2002 | A/New York/179/1999 | A/New York/56/2003 |
| A/New York/119/2002 | A/New York/18/2003 | A/New York/59/2003 |
| A/New York/12/2003 | A/New York/180/2000 | A/New York/6/2004 |
| A/New York/120/2002 | A/New York/181/1999 | A/New York/60A/2003 |
| A/New York/121/2002 | A/New York/182/2000 | A/New York/61A/2003 |
| A/New York/122/2002 | A/New York/185/1999 | A/New York/62A/2003 |
| A/New York/124/2001 | A/New York/186/1999 | A/New York/63/2003 |
| A/New York/127/2001 | A/New York/189/1999 | A/New York/64/2003 |
| A/New York/128/2002 | A/New York/19/2003 | A/New York/65/2003 |
| A/New York/129/2002 | A/New York/192/2003 | A/New York/67/2003 |
| A/New York/13/2003 | A/New York/194/2003 | A/New York/69/2004 |
| A/New York/130/2002 | A/New York/198/2003 | A/New York/7/2003 |
| A/New York/131/2001 | A/New York/199/2003 | A/New York/70/2004 |
| A/New York/132/2002 | A/New York/2/2003 | A/New York/71/2001 |
| A/New York/133/2002 | A/New York/202/2003 | A/New York/74/2002 |
| A/New York/134/2002 | A/New York/21/2003 | A/New York/8/2003 |
| A/New York/135/2002 | A/New York/22/2003 | A/New York/80/2001 |
| A/New York/136/2002 | A/New York/23/2003 | A/New York/81/2002 |
| A/New York/137/1999 | A/New York/24/2003 | A/New York/82/2001 |
| A/New York/138/1999 | A/New York/25/2003 | A/New York/83/2001 |
| A/New York/14/2003 | A/New York/26/2003 | A/New York/84/2001 |
| A/New York/143/1999 | A/New York/27/2003 | A/New York/85/2001 |
| A/New York/147/1999 | A/New York/28/2003 | A/New York/86/2002 |
| A/New York/148/2000 | A/New York/29/2003 | A/New York/87/2002 |
| A/New York/149/1999 | A/New York/3/2003 | A/New York/88/2002 |
| A/New York/15/2003 | A/New York/30/2003 | A/New York/89/2002 |
| A/New York/150/2000 | A/New York/31/2004 | A/New York/90/2002 |
| A/New York/151/1999 | A/New York/32/2003 | A/New York/91/2002 |
| A/New York/152/2000 | A/New York/33/2004 | A/New York/92/2002 |
| A/New York/154/2000 | A/New York/34/2003 | A/New York/93/2002 |
| A/New York/157/1999 | A/New York/35/2003 | A/New York/94/2001 |
| A/New York/158/2000 | A/New York/36/2003 | A/New York/95/2002 |
| A/New York/16/2003 | A/New York/38/2003 | A/New York/96/2002 |
| A/New York/160/2000 | A/New York/39/2003 | A/New York/99/2002 |

**2. PB2** **Background Isolates**

| A/swine/Hong_Kong/121 | A/swine/Hong_Kong/928 | A/swine/Hong_Kong/436 |
| --- | --- | --- |
| A/swine/Hong_Kong/411 | A/swine/Hong_Kong/984 | A/swine/Hong_Kong/q06 |
| A/swine/Hong_Kong/74 | A/swine/Hong_Kong/312 | A/Swine/Hong_Kong/240 |
| A/swine/Hong_Kong/131 | A/swine/Hong_Kong/722 | A/Swine/Hong_Kong/242 |

**3. PB1** **Background Isolates**

| A/swine/Hong_Kong/121 | A/swine/Hong_Kong/928 | A/swine/Finisterre/12 |
| --- | --- | --- |
| A/swine/Hong_Kong/411 | A/swine/Hong_Kong/984 | A/swine/Hong_Kong/436 |
| A/swine/Hong_Kong/74 | A/swine/Hong_Kong/312 | A/swine/Hong_Kong/q06 |
| A/swine/Hong_Kong/131 | A/swine/Hong_Kong/722 |  |

4. PA Background Isolates

| A/swine/Hong_Kong/121 | A/swine/Hong_Kong/928 | A/swine/Finisterre/12 |
| --- | --- | --- |
| A/swine/Hong_Kong/411 | A/swine/Hong_Kong/984 | A/swine/Hong_Kong/436 |
| A/swine/Hong_Kong/74 | A/swine/Hong_Kong/312 | A/swine/Hong_Kong/q06 |
| A/swine/Hong_Kong/131 | A/swine/Hong_Kong/722 |  |

5. HA Background Isolates

| A/Townsville/1/1999 | A/Zhejiang/80/2003 | A/France/27/2000 |
| --- | --- | --- |
| A/Canada/33312/1999 | A/Zhejiang/81/2003 | A/France/3/2000 |
| A/Mar del Plata/267/1999 | A/Zhejiang/92/2003 | A/France/40/2000 |
| A/Santa Fe/466/1999 | A/Zhejiang/95/2003 | A/France/42/2000 |
| A/Virginia/21735/1999 | A/Cheju/274/2002 | A/France/47/2000 |
| A/Malmoe/1/2000 | A/Cheju/311/2002 | A/France/5/2000 |
| A/Oslo/6391/2000 | A/Cheonnam/323/2002 | A/France/50/2000 |
| A/Stockholm/2/2000 | A/Cheonnam/338/2002 | A/France/54/2000 |
| A/Sydney/118/2000 | A/Cheonnam/340/2002 | A/Gothenburg/1/1999 |
| A/Umea/2/2000 | A/Cheonnam/432/2002 | A/Greece/109/1999 |
| A/Chaco/R538/2001 | A/Daejeon/258/2002 | A/Greece/132/1999 |
| A/Cordoba/1007333/2001 | A/Kwangju/219/2002 | A/Hong Kong/1144/1999 |
| A/Neuquen/1016002/2001 | A/Netherlands/1/2002 | A/Hong Kong/1179/1999 |
| A/Fujian/411/2002 | A/Netherlands/120/2002 | A/Inchon/81/1999 |
| A/Ishikawa/102/2002 | A/Ningbo/25/2002 | A/Indiana/28170/1999 |
| A/Ningbo/17/2002 | A/Oslo/669/2002 | A/Kwangju/105/1999 |
| A/Zhejiang/11/2002 | A/swine/Hong Kong/1212/2002 | A/Kwangju/115/1999 |
| A/Christchurch/28/2003 | A/swine/Hong Kong/411/2002 | A/Malmo/2/1999 |
| A/South Australia/39/2003 | A/swine/Hong Kong/74/2002 | A/Mendoza/135/1999 |
| A/Sydney/015/2003 | A/Texas/131/2002 | A/Michigan/22568/1999 |
| A/Wyoming/3/2003 | A/Buenos Aires/722/2001 | A/Michigan/22692/1999 |
| A/Denmark/17-2/2003 | A/Netherlands/118/2001 | A/Moscow/10/1999 |
| A/Denmark/18-2/2003 | A/Netherlands/124/2001 | A/Netherlands/301/1999 |
| A/Denmark/37/2003 | A/Netherlands/126/2001 | A/New Caledonia/1/1999 |
| A/Denmark/13/2003 | A/Neuquen/1038288/2001 | A/Oslo/2512/1999 |
| A/Denmark/92/2003 | A/Neuquen/2260/2001 | A/Oslo/5631/1999 |
| A/Denmark/19/2003 | A/swine/Hong Kong/1311/2001 | A/Panama/2007/1999 |
| A/Denmark/52/2003 | A/swine/Hong Kong/9285/2001 | A/Pusan/71/1999 |
| A/Denmark/70/2003 | A/swine/Hong Kong/9840/2001 | A/Santa Fe/9/1999 |
| A/Denmark/61/2003 | A/Netherlands/3/2000 | A/Stockholm/1/1999 |
| A/Denmark/10/2003 | A/Oslo/841/2000 | A/Stockholm/10/1999 |
| A/Denmark/63/2003 | A/South Africa/40/2000 | A/Stockholm/12/1999 |
| A/Denmark/24/2003 | A/South Africa/96/2000 | A/Stockholm/13/1999 |
| A/Denmark/32/2003 | A/Stockholm/3/2000 | A/Stockholm/8/1999 |
| A/Charlottesville/03/2004 | A/Stockholm/7/2000 | A/Swine/Colorado/23619/1999 |
| A/Nepal/1664/2004 | A/Stockholm/8/2000 | A/swine/Finistere/127/1999 |
| A/Nepal/1717/2004 | A/swine/Hong Kong/312/2000 | A/swine/Hong Kong/4361/1999 |
| A/Nepal/1723/2004 | A/swine/Hong Kong/7220/2000 | A/swine/Hong Kong/q066/1999 |
| A/swine/Guangdong/6/2004 | A/Athens/135/1999 | A/Tehran/83/1999 |
| A/Wellington/1/2004 | A/Buenos Aires/M14/1999 | A/United Kingdom/26554/1999 |
| A/Netherlands/20/2003 | A/Buenos Aires/M6/1999 | A/United Kingdom/34300/1999 |
| A/Netherlands/22/2003 | A/Charlottesville/10/1999 | A/Utah/20997/1999 |
| A/Netherlands/222/2003 | A/Charlottesville/73/1999 | A/Virginia/21754/1999 |
| A/Ningbo/198/2003 | A/Darwin/2/1999 | A/Virginia/21817/1999 |
| A/Ningbo/217/2003 | A/Finland/638/1999 | A/Virginia/21822/1999 |
| A/swine/Guangdong/1/2003 | A/Finland/683/1999 | A/Zhejiang/12/1999 |
| A/swine/Guangdong/3/2003 | A/Finland/695/1999 | A/Virginia/21743/1999 |
| A/Zhejiang/102/2003 | A/Finland/697/1999 |  |
| A/Zhejiang/78/2003 | A/France/23/2000 |  |

6. NP Background Isolates

| A/swine/Hong_Kong/121 | A/swine/Hong_Kong/984 | A/Sydney/274/2000 |
| --- | --- | --- |
| A/swine/Hong_Kong/411 | A/Christchurch/39/20 | A/swine/Finisterre/12 |
| A/swine/Hong_Kong/74 | A/swine/Hong_Kong/312 | A/swine/Hong_Kong/436 |
| A/swine/Hong_Kong/928 | A/swine/Hong_Kong/722 | A/swine/Hong_Kong/q06 |

7. NA Background Isolates

| A/Denmark/13/2003 | A/Montreal/MTL169/2000 | A/Swine/Ohio/891/2001 |
| --- | --- | --- |
| A/Denmark/62/2003 | A/Montreal/MTL63446/2000 | A/Swine/Indiana/P12439/2000 |
| A/Denmark/14-2/2003 | A/Montreal/MTL63/2000 | A/Swine/Minnesota/55551/2000 |
| A/Denmark/19-2/2003 | A/Montreal/MTL152/2000 | A/swine/Hong Kong/1311/2001 |
| A/Denmark/15-2/2003 | A/Montreal/MTL327/2000 | A/swine/Hong Kong/9840/2001 |
| A/Denmark/18-2/2003 | A/Montreal/MTL72692/2000 | A/swine/Hong Kong/9285/2001 |
| A/Denmark/37/2003 | A/Montreal/MTL50/2000 | A/Charlottesville/03/2004 |
| A/Denmark/39/2003 | A/Montreal/MTL512/2000 | A/Netherlands/126/2001 |
| A/Denmark/70/2003 | A/Montreal/MTL20/2000 | A/1496/2002 |
| A/Denmark/10/2003 | A/Montreal/MTL71973/2000 | A/Egypt/96/2002 |
| A/Denmark/63/2003 | A/Montreal/MTL71045/2000 | A/England/57/2002 |
| A/Denmark/60/2003 | A/Montreal/MTL63963/2000 | A/Egypt/21181/2001 |
| A/Denmark/24/2003 | A/Montreal/MTL8/2000 | A/Egypt/84/2001 |
| A/Denmark/32/2003 | A/Montreal/MTL63149/2000 | A/England/627/2001 |
| A/Moscow/10/1999 | A/Montreal/MTL63748/2000 | A/England/691/2001 |
| A/Ireland/10586/1999 | A/Wyoming/3/2003 | A/Yokohama/22/2002 |
| A/Latvia/1097/2000 | A/Pusan/504/2002 | A/Nevada/5/2001 |
| A/Perugia/7/2000 | A/Kyongbuk/320/2002 | A/Oman/16353/2001 |
| A/Valladolid/4/2001 | A/Kwangju/243/2002 | A/Singapore/63/2001 |
| A/Lyon/1242/2000 | A/Kwangju/219/2002 | A/Singapore/66/2001 |
| A/Philippines/26/1999 | A/Daejeon/390/2002 | A/Singapore/73/2001 |
| A/Panama/2007/1999 | A/Daejeon/258/2002 | A/India/77251/2001 |
| A/Hong Kong/6/1999 | A/Cheonnam/340/2002 | A/Norway/70/2005 |
| A/Perugia/5/1999 | A/Cheonnam/338/2002 | A/Argentina/126/2004 |
| A/Hong Kong/2/2000 | A/Cheju/311/2002 | A/Brazil/1759/2004 |
| A/Hong Kong/1789/2000 | A/Swine/Illinois/100084/2001 | A/Hong Kong/2982/2004 |
| A/Hong Kong/221/1999 | A/Swine/Illinois/100085A/2001 | A/Japan/120/2004 |
| A/Montreal/MTL63286/2000 | A/Swine/North Carolina/93523/01 | A/Norway/807/2004 |
| A/Montreal/MTL516/2000 | A/Swine/North Carolina/98225/2001 | A/Singapore/36/2004 |

**7. M1 Background Isolates**

| A/Charlottesville/2003 | A/swine/Hong_Kong/928 | A/Panama/2007/1999 |
| --- | --- | --- |
| A/swine/Hong_Kong/121 | A/swine/Hong_Kong/984 | A/swine/Hong_Kong/436 |
| A/swine/Hong_Kong/411 | A/swine/Hong_Kong/312 | A/swine/Hong_Kong/q06 |
| A/swine/Hong_Kong/74 | A/swine/Hong_Kong/722 |  |
| A/swine/Hong_Kong/131 | A/Moscow/10/1999 |  |

**8. NS1 Background Isolates**

| A/swine/Hong_Kong/121 | A/swine/Hong_Kong/928 | A/Panama/2007/1999 |
| --- | --- | --- |
| A/swine/Hong_Kong/411 | A/swine/Hong_Kong/312 | A/Swine/Finistere/127 |
| A/swine/Hong_Kong/74 | A/swine/Hong_Kong/722 | A/swine/Hong_Kong/436 |
| A/swine/Hong_Kong/131 | A/Moscow/10/1999 |  |
