# Supplementary material for: Whole-Genome Analysis of Human Influenza A Virus Reveals Multiple Persistent Lineages and Reassortment among Recent H3N2 Viruses
Source: PLoS Biol. 2005 Jul 26;3(9):e300. doi: 10.1371/journal.pbio.0030300 (PMC1180517; doi:10.1371/journal.pbio.0030300)
Supplement: Supplementary file 2 [file pbio.0030300.st002.doc]

Supplementary Information 2. Parameter values for maximum likelihood phylogenetic analysis.

**1. New York State Isolates**

PB2

Rate matrix (A-C, A-G, A-T, C-G, C-T, G-T)

1.050485 8.266470 0.742637 4.6400e-13 12.199643 1.000000

Nucleotide frequencies: A=0.34861 C=0.18218 G=0.24148 T=0.22773

Proportion of invariable sites = 0.36624

Shape parameter (alpha) = 0.863797

PB1

Rate matrix (A-C, A-G, A-T, C-G, C-T, G-T)

2.467000 19.069893 1.110727 4.9583e-11 22.285699 1.000000

Nucleotide frequencies: A=0.35789 C=0.19482 G=0.22564 T=0.22165

Proportion of invariable sites = 0.603845

Shape parameter (alpha) = 5.43464

PA

Rate matrix (A-C, A-G, A-T, C-G, C-T, G-T)

2.484844 14.524037 0.988085 0.240314 18.290509 1.000000

Nucleotide frequencies: A=0.34561 C=0.18535 G=0.22990 T=0.23914

Proportion of invariable sites = 0.33327

Shape parameter (alpha) = 0.844998

HA

Rate matrix (A-C, A-G, A-T, C-G, C-T, G-T)

1.267920 8.916698 0.614153 0.155648 8.104141 1.000000

Nucleotide frequencies: A=0.33644 C=0.19795 G=0.22323 T=0.24238

Proportion of invariable sites = 0.520585

Shape parameter (alpha) = 1.35131

NP

Rate matrix (A-C, A-G, A-T, C-G, C-T, G-T)

1.227570 10.800933 0.656722 0.869669 12.978930 1.000000

Nucleotide frequencies: A=0.33890 C=0.19115 G=0.25527 T=0.21468

Proportion of invariable sites = 0.305164

Shape parameter (alpha) = 0.663204

NA

Rate matrix (A-C, A-G, A-T, C-G, C-T, G-T)

1.473288 7.046116 0.530054 0.507467 9.818397 1.000000

Nucleotide frequencies: A=0.31949 C=0.18962 G=0.23287 T=0.25803

Proportion of invariable sites = 0.161264

Shape parameter (alpha) = 0.788905

M1

Rate matrix (A-C, A-G, A-T, C-G, C-T, G-T)

3.271890 18.231068 1.332519 3.1613e-09 22.224933 1.000000

Nucleotide frequencies: A=0.29919 C=0.21620 G=0.26040 T=0.22421

Proportion of invariable sites = 0.696026

Shape parameter (alpha) = 485.389

NS

Rate matrix (A-C, A-G, A-T, C-G, C-T, G-T)

5.144985 48.107405	1.502378 2.286877 32.953960 1.000000

Nucleotide frequencies: A=0.32243 C=0.20197 G=0.24049 T=0.23511

Proportion of invariable sites = none

Shape parameter (alpha) = 1.36502

**2. New York isolates and “background” HA**

Rate matrix (A-C, A-G, A-T, C-G, C-T, G-T)

1.464375 7.171626 0.640236 0.190820 6.940088 1.000000

Nucleotide frequencies: A=0.33910 C=0.22384 G=0.20576 T=0.23129

Proportion of invariable sites = 0.312283

Shape parameter (alpha) = 1.06582

**3. New York isolates and “background” NA**

Rate matrix (A-C, A-G, A-T, C-G, C-T, G-T)

1.761630 5.786348 0.555455 0.346705 7.523131 1.000000

Nucleotide frequencies: A=0.32417 C=0.19049 G=0.23046 T=0.25488

Proportion of invariable sites = 0.180522

Shape parameter (alpha) = 0.766916
